# *Pulsatilla chinensis* extract alleviate *Staphylococcus aureus* induced mastitis in mice by regulating the inflammatory response and gut microbiota

**DOI:** 10.3389/fvets.2025.1603107

**Published:** 2025-05-14

**Authors:** Yifei Xiang, Ziyang Li, Chengzhi Liu, Zhifei Wei, Xuelian Mo, Yawen Zhong, Ruini He, Zhengmin Liang, Yucheng He, Jiakang He

**Affiliations:** ^1^College of Animal Science and Technology, Guangxi University, Nanning, China; ^2^Guangxi Key Laboratory of Animal Breeding, Disease Control and Prevention, Nanning, China; ^3^Guangxi Zhuang Autonomous Region Engineering Research Center of Veterinary Biologics, Nanning, China

**Keywords:** subclinical mastitis, *Pulsatilla chinensis* extract, *Staphylococcus aureus*, mouse mastitis model, gut microbiota

## Abstract

**Introduction:**

Subclinical mastitis (SCM) caused by *Staphylococcus aureus* (*S. aureus*) is widely prevalent in cattle herds around the world, causing huge losses to the dairy cattle farming industry and dairy product production. Currently, the use of hormones and antibacterial drugs is the most effective treatment method. However, issues such as the increase in drug resistance and residues in dairy products limit their further application. In this study, based on the response surface optimization method, *Pulsatilla chinensis* extract (PCE) was prepared from *Pulsatilla chinensis* using ethanol as the medium in a simple, efficient and low-cost way. Its functions were verified both *in vitro* and *in vivo*.

**Methods and results:**

Through the Oxford cup method, MIC/MBC and co-culture experiments, it was demonstrated that PCE had a good inhibitory effect on the proliferation of four strains of *S. aureusin vitro*. The *in vivo* toxicity evaluation proved that PCE had high oral safety. In addition, we screened and established a mastitis model platform for lactating mice to evaluate the expected *in vivo* effects of PCE. The results showed that pre-treatment with PCE for 7 days significantly reduced the bacterial load and the levels of inflammatory factors (IL-6, IL-1β, TNF-α, MPO) in the mammary gland and blood induced by *S. aureus*, improved the pathological damage of the mammary gland tissue, and alleviated the occurrence of mastitis in mice by regulating the intestinal microbiota.

**Conclusion:**

These results verify that PCE can be used to treat mastitis caused by *S. aureus*, and thus it is expected to become an excellent alternative to hormones and antibacterial drugs.

## Graphical abstract

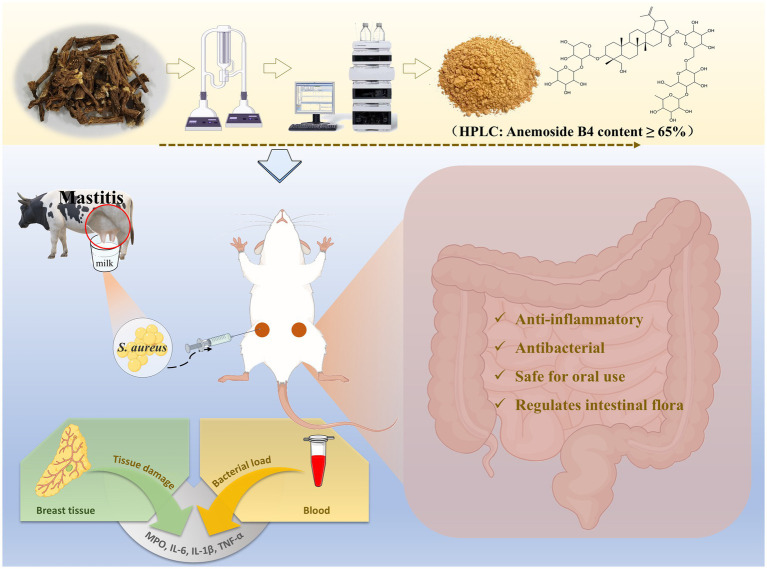

## 1 Introduction

Subclinical mastitis (SCM) is a major industrial bottleneck that restricts the development of the global dairy cow breeding industry and the dairy product industry (1–3). Owing to the lack of obvious symptoms, it can continuously spread among herds and is not easily detected, which consequently leads to a reduction in milk production, a decline in milk quality, culling of cows, and the occurrence of secondary diseases (4). According to incomplete statistics, the economic losses caused by SCM worldwide amount to as many as 35 billion US dollars every year (5, 6).

Infection by pathogenic microorganisms is an important cause of SCM (5, 7). On the one hand, pathogenic bacteria invade the mammary gland, multiply in large numbers and launch attacks, which then trigger an outbreak of inflammation in the mammary region and even systemic inflammatory responses (8). On the other hand, an imbalance in the intestinal flora further exacerbates the difficulty in treating SCM (1, 7). *Staphylococcus aureus* (*S. aureus*) is globally recognized and the most common pathogen for SCM (9), accounting for 40% or more of the available mastitis data (10, 11). Owing to its characteristics, such as extremely high infectivity and long disease course, preventing and controlling dairy cow mastitis has always been difficult. Hormones and antibacterial drugs are the primary solutions for SCM (12). However, long-term or irrational use has led to multiple instances of drug resistance in bacteria, drug residues in milk, and imbalances in the intestinal flora (13, 14), which seriously threaten human public health (dairy products) (15, 16). Therefore, finding new strategies or substitutes for the prevention and treatment of SCM has become a major requirement for overcoming the bottleneck in the development of the dairy cow breeding industry and the dairy product industry (5).

Natural herbal medicines have gradually become important alternative strategies for disease prevention and control because of their advantages, such as high safety, low tendency to generate drug resistance, and multitarget prevention and treatment (17). The development of drugs for the prevention and treatment of dairy cow mastitis from natural medicinal resources has become a new direction for the healthy breeding of dairy cows. *Pulsatilla chinensis* has multiple pharmacological effects, such as anti-inflammatory, antioxidant, and antipathogenic effects (18). However, the antibacterial ability of *Pulsatilla chinensis* and its effect on dairy cow mastitis remain unclear.

In this study, we obtained *Pulsatilla chinensis* extract (PCE) with a relatively high content through a minimalist extraction process. We first demonstrated that PCE has a good inhibitory effect on the proliferation of *S. aureus in vitro*. Through the establishment of a mouse mastitis model during the lactation period, the antibacterial, anti-inflammatory, and mammary gland damage-alleviating effects of PCE *in vivo* were subsequently verified. Finally, the beneficial effect of PCE on the coordination of the intestinal flora was emphasized. This study provides a safer and beneficial alternative treatment strategy for the prevention and treatment of SCM and other diseases caused by *S. aureus*.

## 2 Materials and methods

### 2.1 Ethics statement

The SPF experimental KM mice were all sourced from the Laboratory Animal Center of Guangxi Medical University (Certificate of Conformity: scxk (Gui) 2020–0003). All experiments were approved and supported by the Animal Research Ethics Committee of Guangxi University (Approval No. GXU-2022-326). All animal experiments were carried out in accordance with the “Guidelines for the Care and Use of Laboratory Animals” published by the National Institutes of Health.

### 2.2 Process optimization and preparation of the PCE

There is a close connection between different extraction factors and the content of the target components to be extracted. First, several portions of *Pulsatilla chinensis* medicinal materials (50 g each) were weighed in parallel. The quality of the medicinal materials was tested and confirmed to meet the requirements; that is, the content of *Anemoside B4* in the medicinal materials was ≥ 4.6%. In accordance with HPLC-SPD 20A (Shimadzu (Shanghai) Global Laboratory Consumables Co., Ltd., Shanghai, China), the following variables were investigated: extraction time (0.5 h, 1 h, 1.5 h, 2 h, 3 h), extraction time (once, twice, three times, four times, five times), ethanol volume (50, 60, 70, 80, 90%), solid–liquid ratio (1:5, 1:10, 1:15, 1:20, 1:25), extraction temperature (50°C, 60°C, 70°C, 80°C, 90°C), and particle size of the medicinal materials (whole plant, decoction pieces, 30 mesh, 50 mesh, 80 mesh). Finally, the extracted liquid was filtered and then concentrated under reduced pressure to 50 mL. After that, the content was detected by HPLC.

The three factors with the most significant influence among the above variables were screened out for the response surface methodology (RSM) experiment. The percentage of *Anemoside B4* in the extract (Y) was taken as the response value of the response surface optimization curve. Design Expert 10 was used to design the experiment. According to the 17 schemes simulated by the three factors and three levels designed in the experiment, a response surface optimization curve analysis experiment was carried out to obtain the optimal extraction process and prepare the PCE for subsequent studies.

### 2.3 *In vitro* investigation of the inhibitory effect of PCE on *Staphylococcus aureus*

Three *S. aureus* model strains, including CMCC (B) 26003 (National Center for Medical Culture Collections, Beijing, China), ATCC 25904, and USA 400 (feed from the Department of Veterinary Pharmacology, Jilin University, Jilin, China), and one *S. aureus* clinical isolate, GXU 2017 (isolated, identified and preserved in Guangxi Key Laboratory of Animal Breeding Disease Control and Prevention, Nanning, China), were used in this *in vitro* study, cultured at a constant temperature of 37°C for 12 h and configured into bacterial suspensions for backup.

#### 2.3.1 Determination of the inhibition zone

Different gradients of bacterial suspensions (1 × 10^6^, 1 × 10^7^, and 1 × 10^8^ CFU/mL) were prepared, and then 100 μL of each bacterial suspension was spread onto the bacterial culture plates. Four Oxford cups were placed on each plate and then injected with 100 μL of PCE solution at concentrations of 0 (control), 31.25, 125 and 500 mg/mL. The antibacterial activity was evaluated by measuring the diameter of the inhibition zone of the PCE against *S. aureus*.

#### 2.3.2 Determination of the minimum inhibitory concentration (MIC) and minimum bactericidal concentration (MBC)

100 μL of *S. aureus* suspension (1 × 10^7^ CFU/mL) was added to each well of a 96-well plate, followed by the addition of different concentrations of PCE solutions such that the final concentration of PCE in each well was 250, 125, 62.5, 31.25, 15.63, 7.81, 3.91, 1.95, 0.98, or 0.49 mg/mL. The plate was incubated at a constant temperature of 37°C for 24 h. The wells with clear liquid were judged as the MIC of PCE against *S. aureus*. Subsequently, 100 μL of the mixture was removed from all the clear wells and spread on culture dishes for further incubation. The lowest concentration at which almost no visible colonies grew on the culture dishes was judged as the MBC.

#### 2.3.3 Growth curve of cocultures of PCE and *Staphylococcus aureus*

Solutions of PCE at concentrations of 1/4 MIC, 1/2 MIC, MIC, 2 MIC, and 4 MIC were cocultured with *S. aureus* (1 × 10^7^ CFU/mL) (at 37°C, 180 rmp). Moreover, a blank control group (TSB) and a positive control group (*S. aureus* + TSB) were set up. Then, 200 μL of the coculture mixture was added at 0, 1, 2, 3, 4, 5, 6, 8, 10, and 12 h, and the optical density (OD) values were measured at 600 nm. Finally, the growth curve of the coculture of PCE and *S. aureus* was plotted on the basis of these measured OD values.

### 2.4 Establishment of a mouse mastitis model

Forty 7-week-old male KM mice and eighty 7-week-old female KM mice were housed in a single mouse cage at a male-to-female ratio of 1:2 and were allowed to mate freely to obtain pregnant female mice. Considering animal welfare, the experiment was carried out on the 15th day after the female mice gave birth (by which time the suckling mice had already acquired the ability to survive independently) (11).

The 80 female mice were divided into two physiological cycles, namely, the lactation period and the weaning period. For the lactation period, the maternal mice were separated from the suckling mice 3 h before the experiment (19), whereas for the weaning period, the maternal mice were separated from the suckling mice 5 days before the experiment. There were 40 female mice in each physiological cycle, and each cycle was randomly divided into a control group (*n* = 8) and four gradient *S. aureus* model groups (*n* = 8, with concentrations of 1 × 10^5^, 1 × 10^6^, 1 × 10^7^, and 1 × 10^8^ CFU/mL). Referencing the previous method and optimizing it (11), female mice were anesthetized after a 12-h fasting period. The breasts and the surrounding skin were disinfected with 75% alcohol. The mammary ducts of the fourth pair of nipples were exposed. A flat-tip microsyringe was used to inject 50 μL of *S. aureus* suspension into the mammary ducts of the mice in the model group, while the same volume of normal saline was injected into those in the Control group. Gentle massage was performed to promote the uniform distribution of the injected liquid. After 24 h, blood and mammary gland tissues were collected and stored at −80°C.

### 2.5 Investigation of the oral safety of PCE

Sixty 5-week-old KM mice (with an equal number of males and females) were provided with free access to water and were fed standard feed according to the standard of 10–15% body weight. Six gradient groups were randomly set up according to the dose of PCE (*n* = 10, with an equal number of females, and the experiment was carried out in separate cages), namely, 0 (Control), 1,250, 2,500, 5,000, 10,000 and 20,000 mg/kg. After a single intragastric administration, the clinical symptoms, food intake, water intake and average weight gain of the mice were continuously observed for 7 days. After the experiment, the mice were sacrificed, and pathological changes in the internal organs were observed. The aim of this study was to investigate the oral safety of PCE.

### 2.6 Control of mastitis in lactating mice

Sixty lactating female mice after giving birth were randomly divided into six groups, namely, the Control group (*n* = 10, continuously gavaged with sterile water for 7 days and then injected with 0.9% NaCl into the milk ducts on the 7th day), the *S. aureus* group (*n* = 10, continuously gavaged with sterile water for 7 days and then injected with 1 × 10^7^ CFU/mL *S. aureus* suspension into the milk ducts on the 7th day), the dexamethasone (DEX) control group (*n* = 10, continuously gavaged with 5 mg/kg of DEX for 7 days and then injected with 1 × 10^7^ CFU/mL *S. aureus* suspension into the milk ducts on the 7th day) (20, 21), and the PCE 100, 200, 400 mg/kg dose groups (*n* = 10, continuously gavaged with different doses of PCE for 7 days and then injected with 1 × 10^7^ CFU/mL *S. aureus* suspension into the milk ducts on the 7th day). The doses of PCE were determined with reference to previous reports and preliminary experiments (22). Blood and mammary gland tissues were collected and stored at −80°C.

### 2.7 Bacterial load

Aseptic procedures were performed with a super clean bench. After the mice were anaesthetized, blood was collected from the eyeballs. The first 3 drops of blood were placed into an anticoagulant tube and diluted 5 times the volume of sterile normal saline. Then, 100 μL of the diluted sample was spread on nutrient agar medium. The mixture was incubated at a constant temperature of 37°C for 24 h, and the bacterial load in the blood was calculated.

Ten milligrams of mammary gland tissue was homogenized with 100 times the volume of sterilized normal saline. One hundred microlitres of the tissue homogenate was spread on nutrient agar medium. The mixture was incubated at a constant temperature of 37°C for 24 h, and the bacterial load in the mammary gland tissue was calculated.

### 2.8 Determination of inflammatory factors

The protocol was performed in accordance with the requirements of the instruction manual. Enzyme-linked immunosorbent assay (ELISA) kits were used to evaluate the levels of interleukin-6 (IL-6), interleukin-1β (IL-1β) and tumor necrosis factor alpha (TNF-*α*) in blood and mammary gland tissues (Shanghai Enzyme Linked Biotechnology Co., Ltd., Shanghai, China).

### 2.9 Investigation of myeloperoxidase (MPO)

In accordance with the requirements of the kit instruction manual, serum and mammary gland homogenates were collected to detect the expression level of myeloperoxidase (MPO) (NanJing JianCheng Bioengineering Institute, Nanjing, China).

### 2.10 Observation and scoring of mammary gland tissue pathology

The isolated mammary gland tissues were rinsed with normal saline to reduce the stagnation of milk and then placed in 10% formalin fixative for 24 h. The tissues were dehydrated with gradient ethanol and then embedded in paraffin blocks. The paraffin blocks containing the tissues were cut into slices with a thickness of 3 μm and incubated at 65°C for 4.5 h. The samples were subsequently dewaxed and rehydrated. Finally, the tissues were stained with dyes, and the slides were mounted. The prepared hematoxylin–eosin (HE)-stained pathological sections were used for histological observation and scoring of the mammary gland tissues (19).

### 2.11 DNA extraction and microbiomic analysis of intestinal microorganisms

The contents of the mouse caecum were collected in sterile freezing tubes, snap-frozen in liquid nitrogen and stored, and total DNA was extracted from the contents of the caecum using a DNA extraction kit (AU46111-96, BioTeke, China). Total DNA was amplified by PCR via the primers 341F/805R (341F: 5′-CCTACGGGNGGCWGCAG-3′; 805R: 5′-GACTACHVGGTATCTAATCC-3′; GACTACHVGGTATCTAATCC-3′). PCR amplification of total DNA was performed via the primers 341F/805R (341F: 5′-CCTACGGNGGCWGCAG-3′; 805R: 5′-CACTACHVGGTATCTAATCC-3′), and the resulting PCR products were purified, quantified and analyzed sequentially. Sequencing services were provided by LC-Bio Technology Co., Ltd., Hangzhou, China.

### 2.12 Data analysis

Each experiment was replicated in triplicate and the data derived therefrom were collected and calculated with Excel. Unless otherwise explicitly stated, all data were tested for normality distribution using GraphPad Prism 10.1.2 (GraphPad Software Inc., La Jolla, CA). In the case of normally distributed data, one-way ANOVA and Tukey’s multiple comparison test were employed to analyze and compare the significance of the experimental results, followed by statistical analysis and graphing. A *p*-value of less than 0.05 was regarded as statistically significant.

## 3 Results

### 3.1 Optimization of the extraction process and preparation of the PCE by response surface methodology

**Figure 1:**
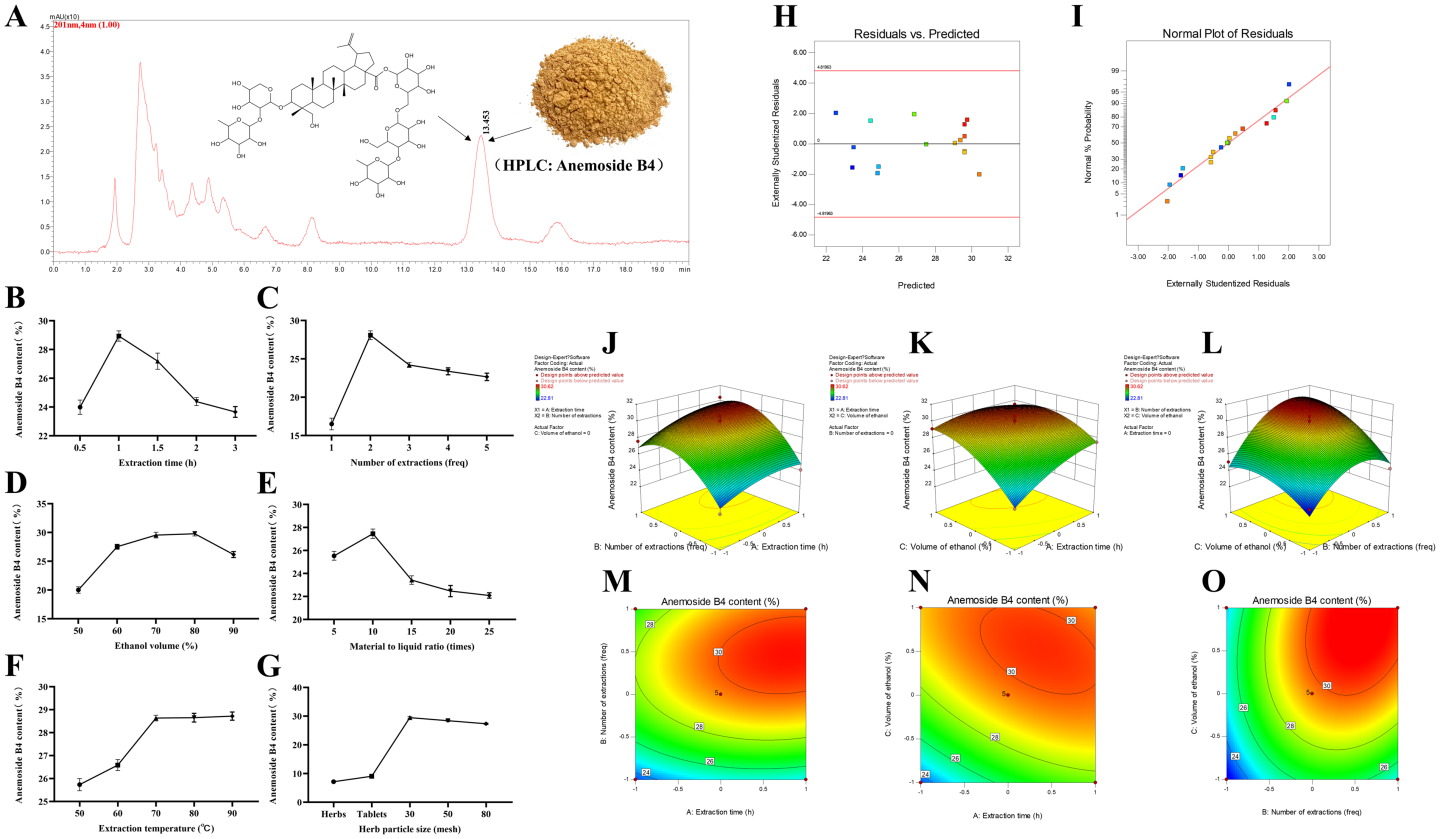
Process optimization and preparation of the PCE. **(A)** HPLC characterization and quantification. The mobile phase for HPLC was methanol and water (64:36), with isocratic elution, a flow rate of 1 mL/min, a column temperature of 30°C, an injection volume of 20 μL and a wavelength of 201 nm. **(B)** Extraction time. **(C)** Extraction times. **(D)** Ethanol volume. **(E)** Solid–liquid ratio. **(F)** Extraction temperature. **(G)** Particle size of medicinal materials. **(H)** Plot of the relationship between residuals and predicted responses. **(I)** Normal distribution plot of residuals. **(J)** Three-dimensional response surface plot of the extraction time (freq) and extraction time (h). **(K)** Three-dimensional response surface plot of ethanol volume (%) and extraction time (h). **(L)** Three-dimensional response surface plot of ethanol volume (%) and extraction time (freq). **(M)** Contour plot of extraction times (freq) and extraction times (h). **(N)** Contour plot of ethanol volume (%) and extraction time (h). **(O)** Contour plot of ethanol volume (%) and extraction time (freq).

Taking the content of *Anemoside B4* in the PCE as the standard, through a climbing test, it was found that the extraction time (Figure 1B), number of extractions (Figure 1C), and volume of ethanol (Figure 1D) had the most significant impacts on the target components in the PCE. Seventeen RSM tests were carried out on the above three factors and the content (%) of *Anemoside B4* (Tables 1, 2).

**Table 1:** Response surface optimization test design scheme.

Level	Factors		
	A: Extraction time (h)	B: Number of extractions (freq)	C: Ethanol volume (%)
−1	0.5	1	60
0	1	2	70
1	1.5	3	80

**Table 2:** RSM multiple regression model design and results.

No.	A: Extraction time (h)	B: Number of extractions (freq)	C: Volume of ethanol (%)	*Anemoside B4* content (%)
1	0	0	0	29.19
2	−1	1	0	27.61
3	−1	0	1	29.11
4	0	1	1	29.66
5	0	0	0	30.04
6	−1	0	−1	23.41
7	0	−1	1	25.09
8	1	−1	0	24.10
9	0	0	0	29.13
10	1	0	1	29.51
11	0	0	0	30.62
12	0	1	−1	24.26
13	0	−1	−1	23.32
14	1	1	0	30.42
15	1	0	−1	27.50
16	−1	−1	0	22.81
17	0	0	0	29.13

Multiple regression analysis yielded a fitting equation (Y = 29.62 + 1.07A + 2.08B + 1.86C + 0.38AB – 0.92 AC + 0.91 BC – 0.79A^2^–2.59B^2^–1.45C^2^). In the analysis of variance, the model was extremely significant (*p* < 0.01), and the lack of fit was not significant (*p* > 0.05) (Table 3), indicating that the results were reasonable and could be used for the analysis and prediction of the PCE content under multiple factors. As seen from the *F* values (Table 3), the number of extractions and the volume of ethanol had relatively large effects on the content of *Anemoside B4* in the PCE. In the plot of residuals versus predicted values, the data points showed a random distribution (Figure 1G), and in the normal probability plot, all the points were distributed on a straight line (Figure 1H), which could accurately reflect the authenticity of the experiment. The RSM is a three-dimensional spatial surface graph composed of three factors, namely, extraction time, the number of extractions, and the volume of ethanol. The steeper the slope is, the more significant the impact (Figures 1J–L). The contour plots all showed irregular ellipses (Figures 1M–O), so the interactions among the three factors were significant.

**Table 3:** Response surface model ANOVA.

Source	Sun of squares	df	Mean square	*F* value	*p*-value Prob > *F*	Salience
Model	122.03	9	13.56	16.28	0.0007	Significant
A-Extraction time	9.22	1	9.22	11.07	0.0126	
B- Number of extractions	34.57	1	34.57	41.50	0.0004	
C-Ethanol volume	27.68	1	27.68	33.23	0.0007	
AB	0.58	1	0.58	0.69	0.4325	
AC	3.40	1	3.40	4.09	0.0829	
BC	3.29	1	3.29	3.95	0.0871	
A^2^	2.65	1	2.65	3.18	0.1176	
B^2^	28.32	1	28.32	34.00	0.0006	
C^2^	8.80	1	8.80	10.57	0.0140	
Residual	5.83	7	0.83			
Lack of fit	3.99	3	1.33	2.89	0.1660	Not significant
Pure error	1.84	4	0.46			
Cor total	127.86	16				

On the basis of the results of the climbing test and RSM, the optimal extraction process for determining the PCE was determined. The content of *Anemoside B4* in the PCE extract was quantified by HPLC as 31.15 (Table 4). One kilogram of *Pulsatilla chinensis* medicinal material was added, 10 times the amount of 75% ethanol was added, hot reflux extraction was performed at 80°C for 70 min, suction filtration was carried out, and the filtrate was temporarily preserved. The filtrate was extracted three times, combined, and then concentrated under reduced pressure to 1 L. After vacuum drying (at 70°C and 0.095 Mpa), it was processed into powder to obtain 255 g of PCE. Then, 1 g of PCE was equivalent to 4 g of the original medicinal material, and the content of *Anemoside B4* in the PCE powder was measured to be 65.17%.

**Table 4:** Process validation results.

No.	*Anemoside B4* content (%)	Average content (%)
1	31.00	
2	31.31	31.15
3	31.12	

### 3.2 *In vitro* bacteriostatic capacity of the PCE

**Figure 2:**
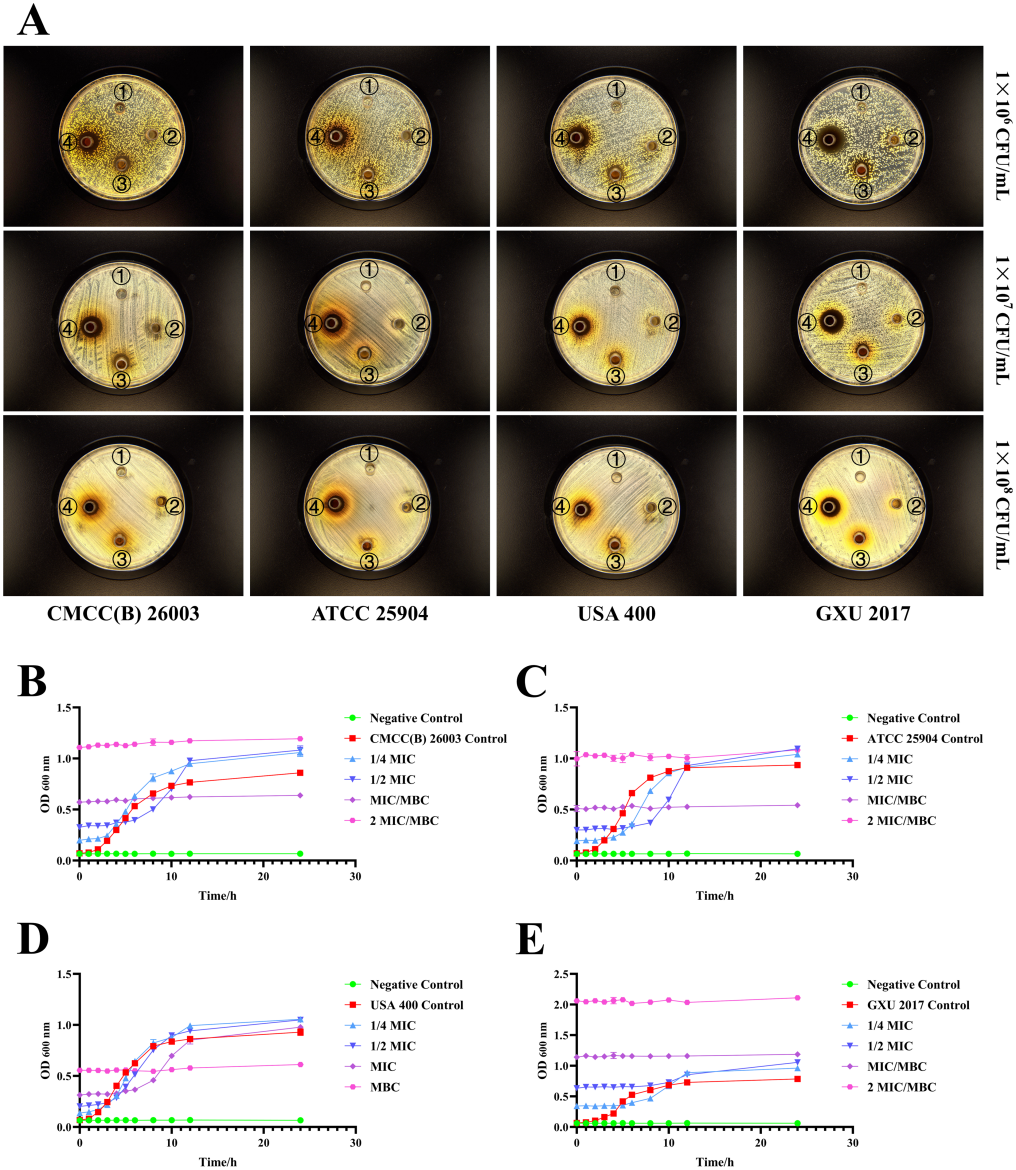
*In vitro* bacterial inhibition ability of PCE. **(A)** Inhibitory circle of the PCE against four kinds of *S. aureus*. The serial numbers ①, ②, ③, and ④ in the Petri dish represent the concentrations of the PCE at 0 (control), 31.25, 125, and 500 mg/mL, respectively. **(B)** Coculture curve of the PCE with the *S. aureus* model strain CMCC(B) 26003. **(C)** Coculture curve of the PCE with the *S. aureus* model strain ATCC 25904. **(D)** Coculture curve of the PCE with the *S. aureus* model strain USA 400. **(E)** Coculture curve of the PCE with the clinical isolate of *S. aureus* GXU 2017.

The diameter of the inhibition zone of the PCE against different *S. aureus* strains was investigated by the Oxford cup method (Figure 2A). When the concentration of *S. aureus* was 1 × 10^7^ CFU/mL, the antibacterial effect was better, and the edge of the inhibition zone was clear. Therefore, a concentration of 1 × 10^7^ CFU/mL was selected for subsequent studies. For the determination of the MIC and the MBC, the MIC values of PCE against *S. aureus* (1 × 10^7^ CFU/mL) CMCC (B) 260,003, ATCC 25904, USA 400 and GXU 2017 were 31.25, 31.25, 15.625 and 62.5 mg/mL, respectively, and the MBC values were 31.25, 31.25, 31.25 and 62.5 mg/mL, respectively. The results revealed that when PCE was cocultured with *S. aureus*, it effectively inhibited the growth of different strains of *S. aureus* within 8 h (Figures 2B–E). The above results indicated that PCE exhibited favorable anti-*S. aureus* effects *in vitro*.

### 3.3 Establishment of a model of mastitis in *Staphylococcus aureus*-infected mice

**Figure 3:**
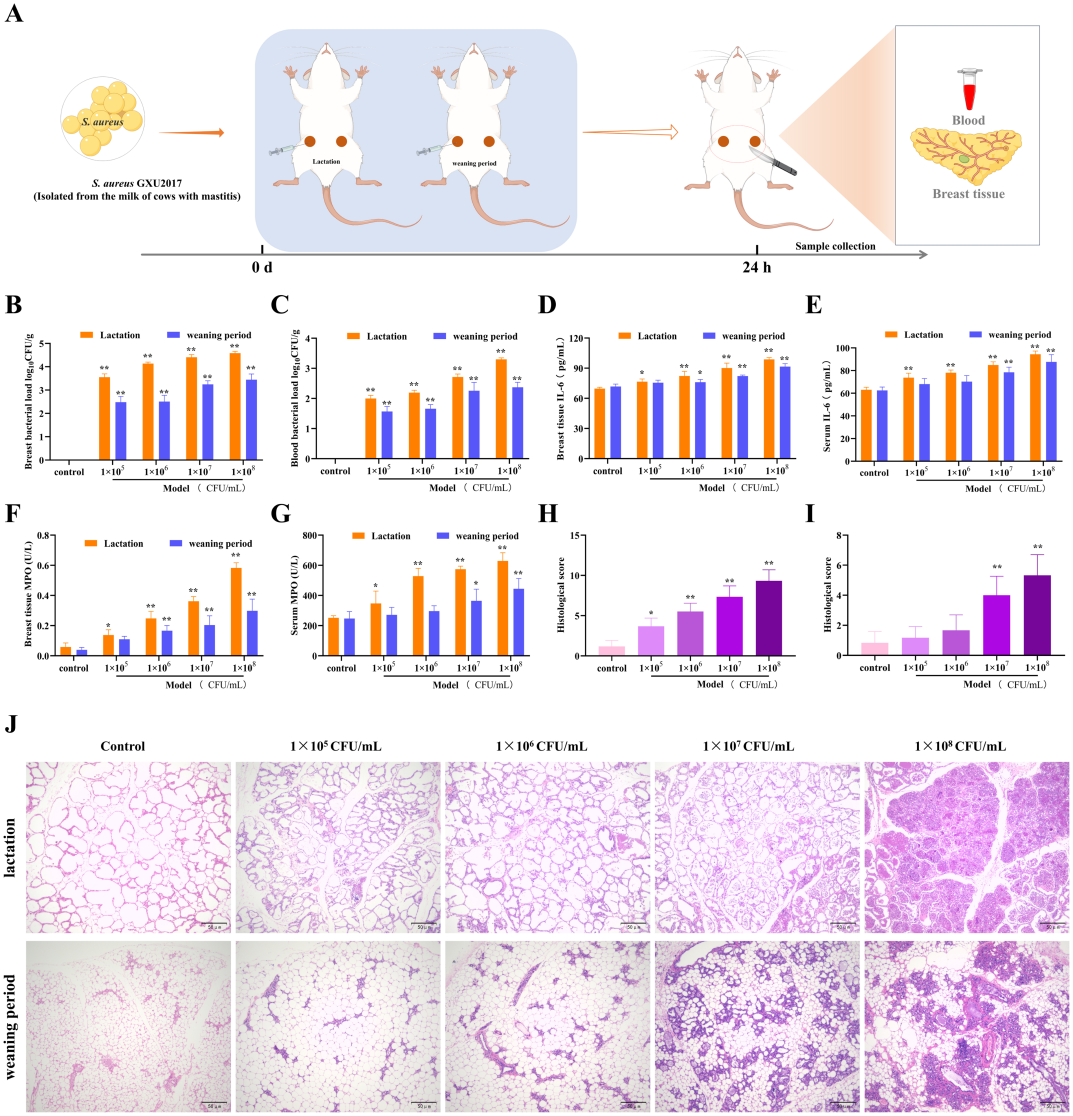
Establishment of the mouse mastitis model. **(A)** Schematic diagram of the establishment of the mouse mastitis model. **(B,C)** The load of *S. aureus* in mammary gland tissue **(B)** and blood **(C)**, 24 h after *S. aureus* infection, mice were anaesthetised and blood was collected from the eyeballs. The first three drops of blood were placed in an anticoagulant tube and diluted to obtain a whole blood dilution. In addition, 10 mg of mammary tissue was homogenized in 100 times sterile saline, and a tissue homogenate was obtained. Then, 100 μL of the whole blood dilution and tissue homogenate was coated on culture medium and incubated at 37°C for 24 h for colony counting and calculation of the bacterial load. **(D,E)** The expression of IL-6 in mammary gland tissue **(D)** and serum **(E)**. **(F,G)** The expression of MPO in mammary gland tissue **(F)** and serum **(G)**. **(H,I)** Pathological scores of mammary gland tissue of mice in the lactation period **(H)** and the weaning period **(I)**. **(J)** Pathological sections of mammary gland tissue of mice in the lactation and weaning periods (HE, ×100).

To investigate the protective effect of PCE on mastitis, we examined mice in two physiological cycles, namely, the lactation and weaning periods, and established a mastitis model on the basis of their sensitivity to invasion by different concentrations of *S. aureus* (GXU2017) (Figure 3). Compared with those in the weaning period, the bacterial loads in both mammary gland tissues (Figure 3B) and blood (Figure 3C), which exhibited a concentration-dependent pattern, were greater in the lactating period than in the weaning period, and the bacterial load in mammary glands was much greater than that in blood. Compared with those in the control group, the expression levels of IL-6 (Figures 3D,E) and MPO (Figures 3F,G) in the serum and mammary gland tissues of lactating mice were significantly greater (*p* < 0.01). In contrast, the expression of these indicators in weaning mice was significant only under high-concentration bacterial infection. Histological analysis demonstrated that (Figure Compared with those in the weaning period), the mammary gland tissue of lactating mice presented larger-volume acini, thicker acinar walls and a more complete honeycomb structure, more pronounced inflammatory cell infiltration, acinar wall hyperplasia and more severe pathological alterations. In conclusion, an experimental surrogate model of dairy cow mastitis was established by infecting lactating mice with 1 × 10^7^ CFU/mL *S. aureus* for 24 h.

### 3.4 Oral safety of the PCE

**Figure 4:**
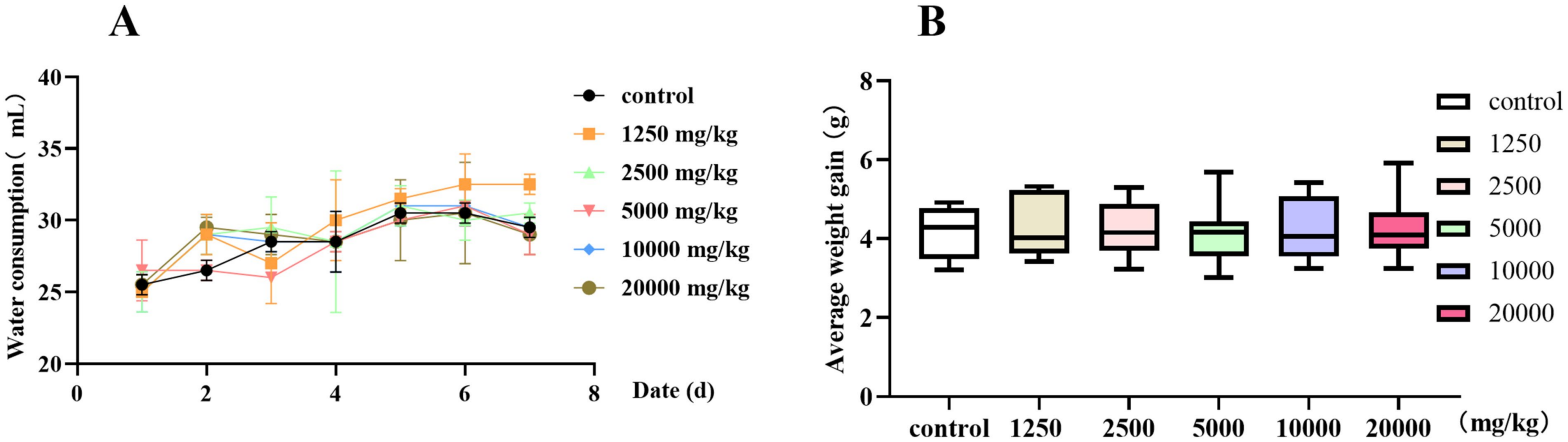
Oral safety of PCE in mice. **(A)** Litter size and water intake. **(B)** Average body weight.

Before a drug is taken orally, its safety needs to be determined. Therefore, we conducted a 7-day oral safety evaluation. When the oral concentration of PCE was less than 20,000 mg/kg, there was no significant difference (*p* > 0.05) in water intake (Figure 4A) or weight gain (Figure 4B) between the test groups and the control group. No death or poisoning phenomenon occurred, and the mice were in a good mental state with a normal appetite and thirst. No obvious pathological changes were found in the internal organs during necropsy. In summary, PCE has excellent oral safety *in vivo*.

### 3.5 Protective effect of PCE against mastitis in female mice

**Figure 5:**
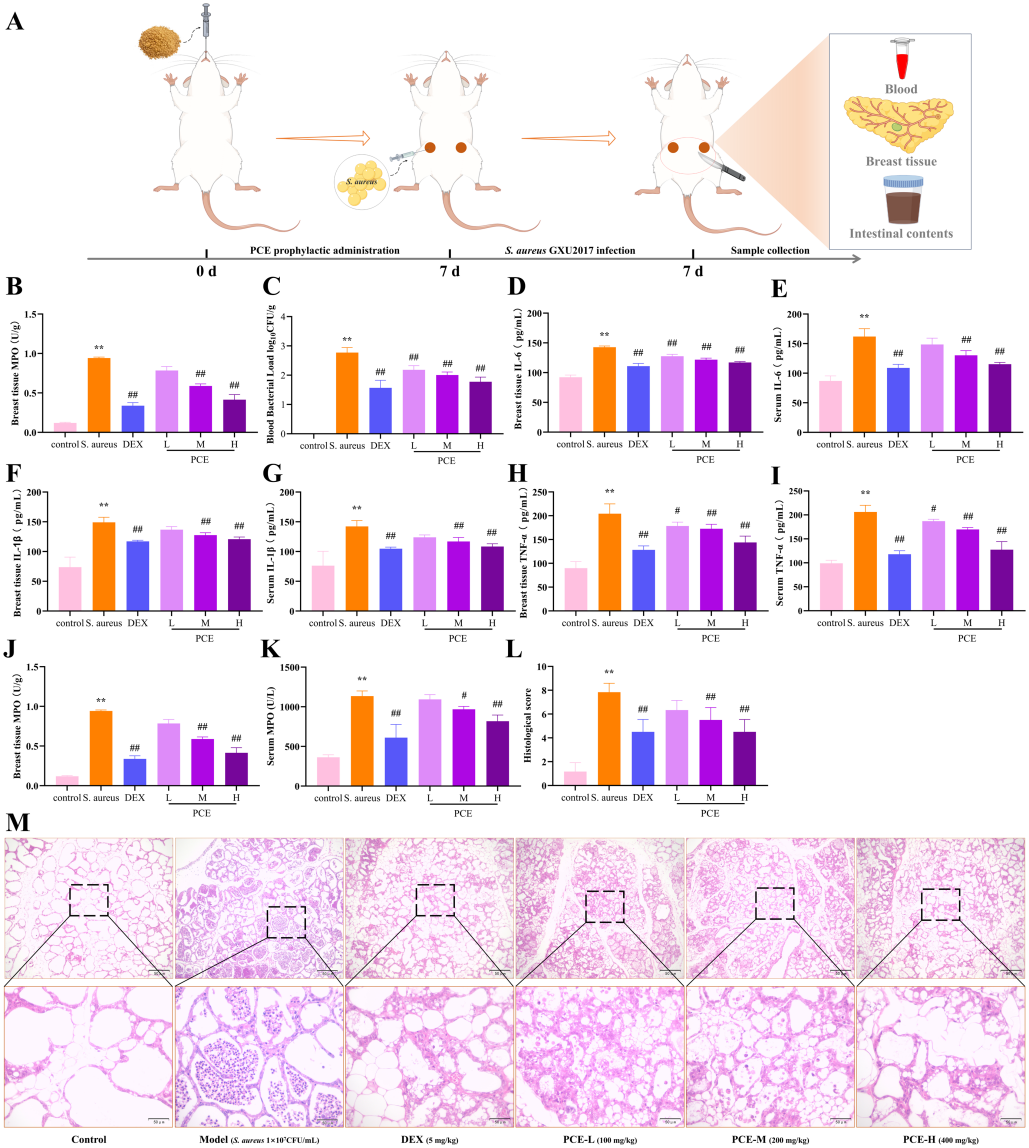
Protective effect of PCE against mastitis in female mice. **(A)** Model diagram of the experimental procedure. **(B,C)** Bacterial load in mammary tissue and blood. **(D,E)** Mammary tissue and serum IL-6 expression levels. **(F,G)** Mammary tissue and serum IL-1*β* expression levels. **(H,I)** Breast tissue and serum TNF-*α* expression levels. **(J,K)** Breast tissue and serum MPO expression levels. **(L)** Histopathological score. **(M)** Breast histological sections (HE, ×100, ×400). * and # indicate comparisons with the blank control and model groups, respectively, and *p* < 0.05 was considered statistically significant.

To evaluate the effect of PCE in the mouse mastitis model, *S. aureus* was inoculated into the mammary ducts after continuous intragastric administration of PCE for 7 days (Figure 5A). Studies have shown that pretreatment with PCE (100, 200, or 400 mg/kg) can significantly reduce the excessive expression of bacteria (Figures 5B,C) and inflammatory factors (IL-6, IL-1β, TNF-*α* and MPO) (Figures 5D–K) in mammary gland tissues and blood (*p* < 0.01). Histological analysis revealed that the invasion of *S. aureus* into the mammary gland damaged the tissue structure of the mammary gland and induced a large number of infiltrating inflammatory cells and colloid secretions in the alveolar cavity (Figures 5L,M). In mice pretreated with PCE, the tissue structure of the mammary gland was relatively intact and clear, and only a small number of inflammatory cells adhered to the alveolar walls and around the lobular ducts. Overall, the 200 mg/kg dose had the greatest effect. These results indicate that oral administration of PCE can significantly reduce the bacterial load and inflammatory response in blood and mammary gland tissues induced by *S. aureus* and alleviate damage to mammary gland tissues.

### 3.6 Beneficial effects of PCE on the intestinal flora

**Figure 6:**
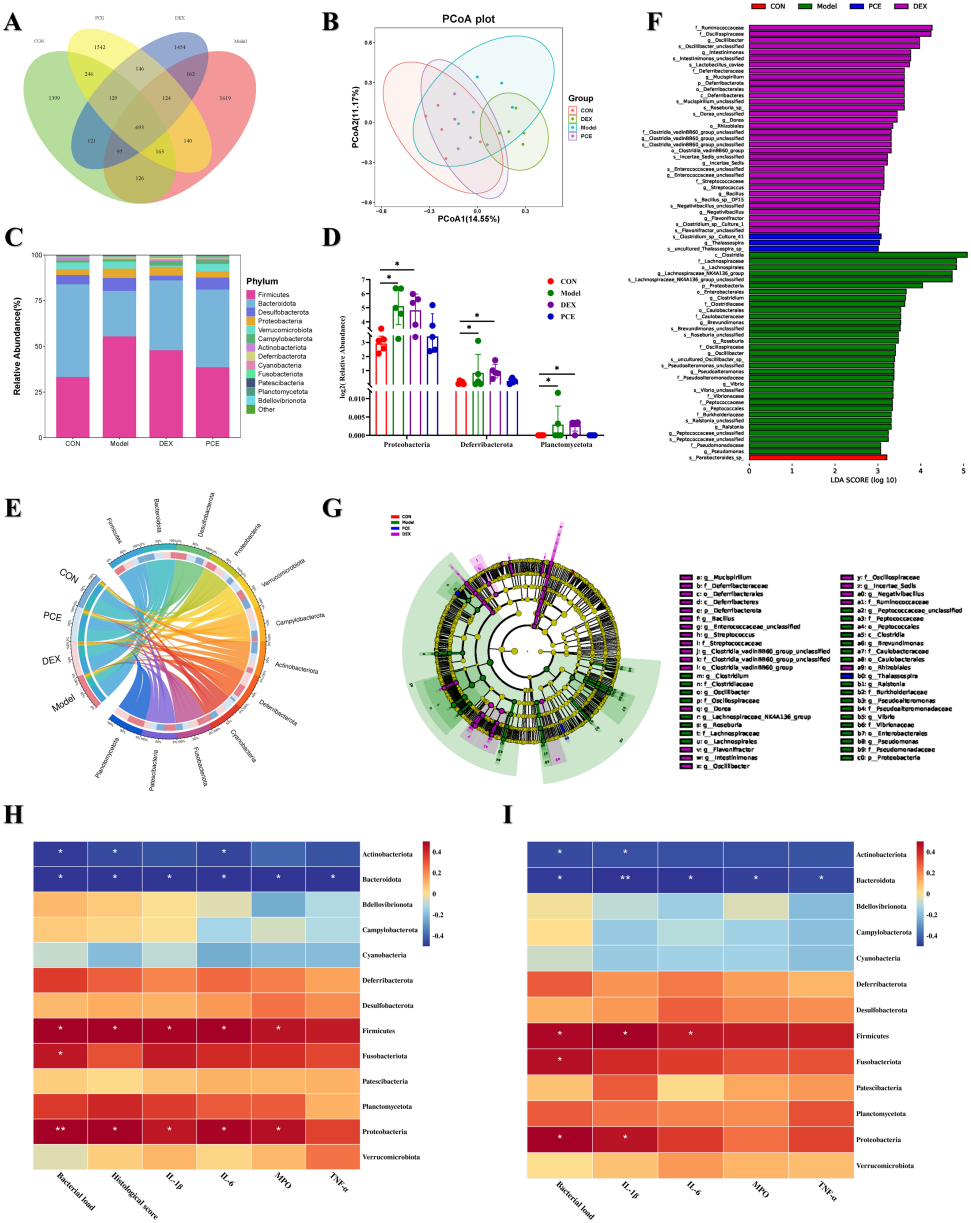
Effects of PCE on the diversity of the gut microbiota in mice. **(A)** Venn diagram of ASV analysis. **(B)** β-diversity analysis diagram (PCoA). **(C)** Stacked bar chart of the gut microbiota at the phylum level. **(D)** Diagram of significant differences in the gut microbiota at the phylum level. **(E)** Proportional and chord diagram of the gut microbiota at the phylum level (the left side shows the grouping information, and the right side shows the top 12 phyla in terms of abundance and their corresponding abundance information). The wider the width is, the greater the abundance is, and the narrower the width is, the lower the abundance is. **(F)** Distribution bar chart of linear discriminant analysis effect size (LEfSe) difference analysis of the gut microbiota (*p* < 0.05). The length of the bars represents the richness of the differential species. **(G)** Evolutionary branching diagram of LEfSe difference analysis of the gut microbiota (*p* < 0.05). The size of the nodes represents the richness of the differential species. **(H,I)** Heatmap analysis of the correlation between the gut microbiota and mammary gland tissue phenotypes **(H)** and blood phenotypes **(I)**. Red indicates a positive correlation, whereas blue indicates a negative correlation. The depth of the color is equivalent to the degree of correlation dependence. The horizontal and vertical coordinates are described previously. * and ** indicate significant differences (*p* < 0.05) and extremely significant differences (*p* < 0.01), respectively, all of which are statistically significant.

We investigated the microbiome of the caecal contents of the mice. Principal coordinate analysis (PCoA) revealed that the microbiota structures of the CON group and the PCE group were relatively similar, while there were significant differences in the microbiota structures between the DEX group and the Model group compared with those of the CON group (Figure 6B). Therefore, it is unreasonable to simply determine whether it is beneficial or harmful to health on the basis solely of the increase or decrease in the number of amplicon sequence variants (ASVs). The number of ASVs in the gut microbiota represented only a quantitative change, as demonstrated by the greater number of ASVs in the DEX group and the Model group (Figure 6A). The species richness at the phylum level is shown in Figure 6C. *Firmicutes* and *Bacteroidota* constituted the main framework of species at the phylum level (accounting for ≥80%). *Firmicutes* are mainly involved in energy acquisition, intestinal homeostasis, nutrient absorption, and immune regulation, while *Bacteroidota* are mainly involved in food digestion, nutrient synthesis, intestinal immunity, and body metabolism. The ratio of the two is considered a key indicator of intestinal health status. They work together to maintain the balance of the intestinal microecology. An imbalance of either one may lead to intestinal dysfunction, which in turn can trigger a variety of health problems (23). A significant difference analysis at the phylum level revealed that, compared with those in the CON group, the relative abundances of *Proteobacteria*, *Deferribacterota* and *Planctomycetota* in the intestines of the Model and DEX groups were significantly greater (Figure 6D). Moreover, the PCE group presented a framework similar to that of the control group at the phylum level. Interestingly, *Planctomycetota* appeared only in the intestines of the mice in the Model group and the DEX group but not in those of the other two groups. The proportional and chord diagrams (Figure 6E) more clearly demonstrated the distribution proportions of the dominant microbiota in each group at the phylum level. In addition, through linear discriminant analysis effect size (LEfSe) analysis, significant differences in the species of the gut microbiota among the different treatment groups of mice were detected (Figures 6F,G). Notably, both the DEX group (purple) and the Model group (green) presented a large number of species with significant differences, and these differences may affect the health of the animals. A correlation clustering-labeled heatmap was used to evaluate the correlations between the gut microbiota and multiple phenotypes in mammary gland tissues (Figure 6H) and blood (Figure 6I). The abundances of *Actinobacteriota* and *Bacteroidota* were more negatively correlated with the relevant phenotypes (*p* < 0.05). Therefore, these microbiota may have inhibited the occurrence of mastitis to some extent. However, the abundances of *Firmicutes*, *Fusobacteriota* and *Proteobacteria* were more positively correlated with the relevant phenotypes (*p* < 0.05), indicating that they promoted the occurrence of mastitis. These differential results are expected to become biomarkers for the clinical diagnosis of mastitis.

## 4 Discussion

Although the pathogenesis of bovine mastitis has become increasingly clear (5, 24), there are still no effective preventive and control measures available at present to alleviate the spread of this disease. Current treatments are still limited to approaches such as hormone and antibacterial drug therapies, supportive (severe cases) treatments, and surgical interventions (25). Surveys have indicated that 60–70% of all hormones and antibacterial drugs on dairy farms are used for the prevention and treatment of mastitis (5). This irrational use of drugs has promoted the emergence of multidrug resistance in bacteria, mainly *S. aureus* (26). Moreover, most chemical drugs are restricted by withdrawal periods, and the spread and infection of bacterial diseases increase significantly during the withdrawal period (27). When the withdrawal period is unstable, drug residues in milk pose a threat to human health and present hidden dangers for disease treatment (28). In the previous few years, the number of human deaths caused by drug resistance exceeded one million cases. Therefore, it has become increasingly important to search for and develop natural alternatives to address diseases with complex aetiologies (29).

Natural plants and their active ingredients are regarded as the most suitable alternative therapies for the treatment of SCM, as they not only have remarkable efficacy but also possess characteristics such as good safety, a low propensity for drug resistance and drug residues, which are beneficial to food safety and human public health (30). *Pulsatilla chinensis* and its active ingredients are well-known natural herbal medicines that have continuously attracted our attention. Our previous studies revealed that *Anemoside B4* could prolong the survival time of mice infected with *Salmonella*, reduce the bacterial load in tissues, ameliorate the inflammatory response induced by Salmonella and alleviate intestinal damage. Moreover, *Anemoside B4* improved the colony structure and diversity of the intestinal microbiota in mice infected with Salmonella and thus restored the balance of the intestinal microbiota (31). *Anemoside B4* was the index component with the highest content in *Pulsatilla chinensis*. Therefore, optimizing the preparation process of PCE is conducive to increasing the content of *Anemoside B4*, which can better demonstrate its clinical application, in-depth processing and pharmacological activities. However, the research and development of veterinary drugs differ from those of human drugs. It needs to be based on economic benefits, and processes that are relatively complicated or have high extraction costs are difficult to apply in actual production (32). In this study, the heating reflux method, which has a relatively low extraction cost, was selected. On the basis of RSM optimization, PCE was finally prepared through techniques such as reflux extraction, vacuum concentration and vacuum thermal drying (Figure 1), and its pharmacological efficacy was evaluated.

Using dairy cows as experimental animals incurs relatively high costs and is not conducive to the implementation of early experiments and methodology. Therefore, research on alternative models for bovine mastitis has become more significant. In this study, we established a model of mastitis in lactating mice induced by *S. aureus*, which greatly reduced the research cost of directly using dairy cows for early clinical trials and improved animal welfare, which has drawn much attention. To gain a clearer understanding of the susceptibility to bacterial invasion under different physiological states, we conducted a comparative study on mice in two physiological cycles, namely, the lactating and weaning periods, with respect to aspects such as the bacterial load, expression of inflammatory factors, and histology. The results revealed that there were significant differences in the histological structure of the mammary gland under different physiological cycles. During the lactating period, the mammary gland was larger in size, with thicker alveolar walls and less connective tissue and adipose tissue, which was more conducive to the infection and colonization of *S. aureus*. With the subsequent invasion of the pathogen, an inflammatory storm subsequently occurs in the mammary tissue and blood. Previously, the most commonly used method was to induce a mastitis model using lipopolysaccharide (LPS) (33). However, LPS is a single commercial endotoxin, and the mastitis induced by it lacks clinical relevance. Pathogenic microorganisms obtained from the milk of cows with mastitis have greater research value. In this study, *S. aureus* (GXU 2017) isolated from the milk of cows with mastitis was used to artificially infect the mammary glands of lactating mice, and a model of mastitis in lactating mice induced by *S. aureus* was established, laying a more solid foundation for the disease model for the subsequent implementation of clinical trials.

The superiority of the PCE was subsequently investigated. *In vitro*, PCE exhibited a good inhibitory effect on the growth and reproduction of four *S. aureus* strains (including three model strains and one clinical isolate). *In vivo*, the bacterial load in the blood demonstrated that *S. aureus* could disrupt the integrity of the blood–milk barrier. After invading the blood through the mammary gland, it induces the recruitment of many neutrophils and further triggers a systemic inflammatory response. The anti-inflammatory and antibacterial effects of PCE and DEX were confirmed in this study. Both of these compounds can reduce the bacterial load in mammary tissue and blood; effectively ameliorate the high expression of inflammatory factors such as MPO, IL-1β, IL-6, and TNF-*α*, as well as pathological damage to the mammary tissue and blood; and verify the oral safety of PCE. The occurrence of mastitis is usually accompanied by the appearance of purulent exudates, and suppurative inflammation is closely related to the defensive response of neutrophils. The increase in neutrophils can relatively intuitively reflect the degree of damage caused by mastitis (34, 35). MPO is present in a certain proportion of neutrophils (34), and its expression level can, to some extent, reflect the degree of neutrophil infiltration and thus reflect the inflammatory situation of the animal body. Moreover, as one of the most typical messengers in the inflammation family (36, 37), the expression levels of IL-1β, IL-6, and TNF-α can be used to better evaluate the damage caused by mastitis (38).

Certainly, in bacterial diseases, the short-term curative effects of hormones and antibacterial drugs are widely recognized. However, numerous studies have already provided compelling evidence regarding the disruption of the gut microbiome by hormones and antibacterial drugs, mainly focusing on aspects such as the composition, diversity, and abundance of the gut microbiota and the reduction in the number of beneficial bacteria (39–41). This is detrimental to the physical health of animals and may serve as a trigger for the next outbreak of disease. Multiple studies have also demonstrated a correlation between the functions of the gut microbiota and mastitis (5, 42, 43). The gut microbiota is an important foundation for maintaining the stability of the gut microenvironment and preventing pathogen invasion (44, 45). Our research has shown that while PCE can effectively alleviate mastitis in mice and the inflammatory response of the body, it can also effectively improve or enhance the beneficial properties of the gut microbiota in mice. Although DEX can effectively suppress the occurrence of the inflammatory response in a short period of time, it is unfavorable for the gut microbiota. The long-term use of DEX may be accompanied by an imbalance in the gut microbiota and even the emergence and increase in harmful bacteria, which reflects the superiority of PCE over DEX. In the intestinal microbiota of animals, the abundances of different phyla are closely associated with the regulation of the host’s inflammatory response. Actinobacteriota and Bacteroidota have been proven to maintain intestinal homeostasis through an anti-inflammatory mechanism mediated by their metabolites. In contrast, the abnormal enrichment of Firmicutes, Fusobacteriota, and Proteobacteria is significantly correlated with the formation of a pro-inflammatory microenvironment (46). The associations among the gut microbiota, mammary tissue, and blood phenotypes have enabled us to understand the beneficial aspects of *Actinobacteriota* and *Bacteroidota*, as well as the unfriendly aspects of *Firmicutes*, *Fusobacteriota*, and *Proteobacteria*. These differentially abundant microbiota may become biomarkers for the clinical diagnosis of bovine mastitis.

## 5 Conclusion

In the posthormone and antibacterial drug era, we have developed an alternative, PCE, which is suitable for clinical production and application. This alternative can effectively inhibit the proliferation of *S. aureus in vitro*. On the premise of oral safety, it has proven its protective ability against *S. aureus*-induced mastitis in mice. Compared with DEX, it can suppress inflammation without disrupting the balance of the intestinal microbiota and there is no risk of drug residues. This study provides an alternative or complementary therapy to hormones and antibacterial drugs for the prevention and treatment of SCM. Of course, more in-depth research is needed to support the process from our current research to the launch of a new drug.

## Data Availability

The original contributions presented in the study are included in the article/supplementary material, further inquiries can be directed to the corresponding authors.
